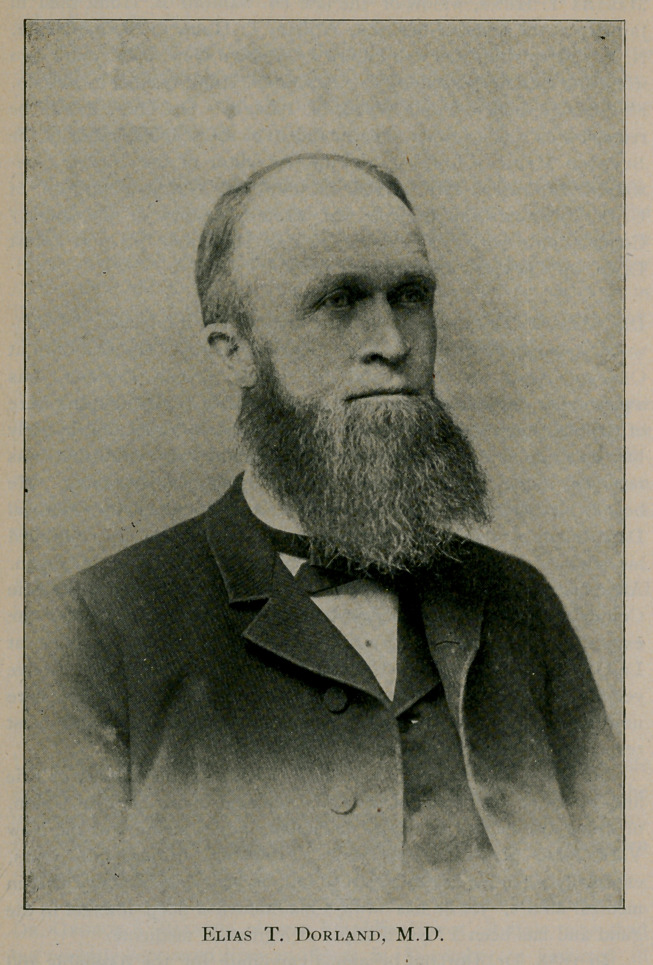# Dr. Elias T. Dorland

**Published:** 1900-03

**Authors:** 


Dr. Elias T. Dorland, of Buffalo, died at his residence, 388 Elmwood Avenue, February 20, 1900, aged 67 years. He was born at Oswego, April 12, 1832, and his father Joseph Dorland, was a prominent physician in that region of the state. His ancestry were of the old Dutch stock, so famous in the Mohawk Valley, but in early life he came to Erie County, where his preliminary education was received in the public schools and at Springville Academy. He began his medical studies in Buffalo and attended the Medical Department of the University for a year. Afterward he continued his studies in and was graduated from the University of Michigan. After his graduation he was appointed resident physician at the Erie County Almshouse, which post he held for two years. After the expiration of his term of service he engaged in private practice at La Grangeville, Dutchess County, where he remained for twelve years. In 1866 he returned to Buffalo and continued to live here until his death. Dr. Dorland was one of the best known and most respected of the older physicians in Buffalo.

He was a member of the Medical Society of the County of Erie and served as its president in 1886; member of the Medical Union, of which also he had served as president; and a member of the New York State Medical Association. However, during late years, especially since his health began to fail, he has taken no active part in medical affairs, yet he has always maintained a deep interest in the guild and has been a loyal disciple of legitimate medicine.

In 1888, Dr. Dorland became a candidate for the Assembly and his successful opponent was William F. Sheehan, who became speaker, and afterward lieutenant governor. He was a mason, a member of the American Legion of Honor, and a member of the Delaware Avenue Baptist Church.

ELIAS T. DORLAND, M.D.

In 1856, Dr. Dorland married Jane C. Congdon, of La Grangeville. He is survived by the widow and one son, an only child,

George E. Dorland. His funeral was largely attended Friday afternoon, February 23d, from the family home at No. 388 Elmwood Avenue, and the interment was at Forest Lawn.

The ceremony was conducted by the Rev. O. P. Gifford, assisted by the Rev. George Whitman. The honorary bearers were Drs. U. C. Lynde, John Hauenstein, Thomas Lothrop, C. C. Wyckoff, A. A. Hubbell, D. W. Harrington, H. R. Hopkins, A. H. Briggs, L. F. Boies, J. B. Coakley, W. G. Gregory and George E. Fell.

The active bearers were Clarence McGregor, N. Preston Taft, Fred K. Hiser, George W. Wilson, Charles C. White, George S. Buck, Joseph H. Wilson and William E. Broughton.

## Figures and Tables

**Figure f1:**